# Realities in cost-effectiveness analyses: a study of castration-resistant prostate cancer patients using a medical claims database

**DOI:** 10.1186/s40064-015-1413-9

**Published:** 2015-10-19

**Authors:** Susumu Kunisawa, Chihiro Tange, Kojiro Shimozuma

**Affiliations:** Department of Biomedical Sciences, Ritsumeikan University, Nojihigashi 1-1-1, Kusatsu, 525-8577 Shiga Japan

**Keywords:** Cost-effectiveness analysis, Prostate cancer, Claims data, Health care costs

## Abstract

Previous cost-effectiveness analyses (CEAs) of abiraterone for castration-resistant prostate cancer (CRPC) patients have not shown favorable results for this new drug. These CEAs were generally conducted based on models used in clinical trials, where comparisons were made with patients given placebos. However, details on any other therapies provided to the comparison groups were not analyzed. These additional therapies should be considered when conducting CEAs to ensure better applications to clinical practice and policymaking. The objective of this study was to elucidate the actual therapies provided to CRPC patients using real-world claims data. We obtained anonymized computerized health care claims data of Japanese prostate cancer patients from the Japan Medical Data Center. This database comprises data from more than 2.5 million insured persons aged below 75 years from over 50 companies between January 2005 and June 2013. From among the prostate cancer patients, we identified CRPC patients as those who had been administered docetaxel, and further investigated their treatments and health care costs. Health care costs were estimated using a regression model accounting for variations in inpatient care, chemotherapies, death, and age. We identified 2138 prostate cancer patients, 36 of whom had been administered docetaxel. We excluded patients diagnosed with other cancers, resulting in a final sample of 18 cases. Of these, 66.7 % were administered other types of chemotherapy, which had not been considered in the control groups in most previous CEAs. We estimated mean health care costs for CRPC to be approximately US$952 per patient per month, and found that these costs were significantly affected by inpatient care and chemotherapy use. Actual therapies include a variety of treatments for CRPC patients, including various types of chemotherapy. Our study estimated health care costs based on real-world claims data. This study contributes to future CEAs by not only providing an estimate of health care costs for these patients, but also demonstrating that the actual therapies provided to comparison groups should be considered when conducting CEAs.

## Background

Due to increasing health care costs, there is growing interest in cost control measures such as the use of generic drugs and efficient resource allocations. Health technology assessments (HTA) are one of the current methods used to guide resource allocations in health care. In general, newly developed drugs shown to be more effective than existing drugs have higher costs, and their uncontrolled introduction into markets can therefore contribute to unilateral increases in health care costs. As a result, HTAs are needed to evaluate whether new drugs and new technologies are sufficiently cost-effective to introduce into current markets.

Cost-effectiveness analyses (CEA) are a major component of HTAs, and are frequently applied to evaluate the introduction of new drugs. The United Kingdom’s National Institute for Health and Care Excellence (NICE) uses this assessment method to evaluate the cost-effectiveness of new drugs and medical procedures. Other countries have also implemented similar approaches, such as the Canadian Agency for Drugs and Technologies in Health (CADTH) and Sweden’s Tandvårds-och läkemedelsförmånsverket (TLV) (http://www.ispor.org/htaroadmaps/). The Japanese government has also shown interest in this assessment method, and is currently planning to introduce CEAs to guide the control of drug and medical device prices, as well as to inform policymaking concerning reimbursements (Ministry of Health, Labour and Welfare 2014a).

In CEAs that compare two procedures (e.g., procedure A with procedure B), the incremental cost-effectiveness ratio (ICER) is widely used as an indicator and is calculated using the formula:

$${\text{ICER}} = \frac{{{\text{Cost A }} - {\text{ Cost B}}}}{{{\text{Effectiveness A}} - {\text{Effectiveness B}}}}.$$

Effectiveness is often calculated using quality-adjusted life years (QALYs). In the above equation, costs refer to health care costs, and cost components can vary according to the perspective of analysis (such as the perspectives of patients or payers).

## Castration-resistant prostate cancer

Although prostate cancer patients generally have low mortality rates, especially in early detection cases (Mohler et al. 2014), treatment for recurrent or castration-resistant prostate cancer (CRPC) cases is considerably more difficult. Japanese guidelines recommended the use of docetaxel to CRPC patients, but there are currently no clear guidelines for treatments after docetaxel use (The Japanese Urological Association 2012). Abiraterone, a 17-α-hydroxylase/C17,20-lyase (CYP17A1) inhibitor, has been recently developed for the treatment of CRPC patients after docetaxel therapy. Its effectiveness has been proven for CRPC and is described in detail elsewhere; briefly, a phase III trial showed significantly longer survival and lower prostate-specific antigen levels in CRPC patients treated with abiraterone after docetaxel when compared with patients treated with a placebo (de Bono et al. 2011). After proof of effectiveness was demonstrated, the cost-effectiveness of abiraterone was investigated. NICE estimated the ICER for abiraterone to be £46,800 (approximately US$50K)/QALY (Dyer et al. 2012), Zhong et al. estimated US$94K/QALY (Zhong et al. 2013), Wilson et al. estimated US$123.4K/QALY (Wilson et al. 2014), and Shibahara et al. estimated US$170K/QALY for Japanese patients (Shibahara et al. 2014). Although a universally accepted threshold amount has yet to be determined, these estimates can be considered high when using an ICER threshold of US$50K/QALY (Neumann et al. 2014), thereby indicating that abiraterone may not be cost-effective.

In all of these studies, the cost-effectiveness of abiraterone was compared with a placebo. It is ostensibly reasonable to use placebos for comparisons partly because there are no other major treatment recommendations for CRPC patients, and also because the original clinical trial had also compared abiraterone with a placebo.

## Real-world data

In recent years, data have become increasingly computerized, and real-world data extracted from existing databases have attractive research applications. For example, these data have potential applications as a source of health care cost data for CEAs. The Japanese government’s drive to computerize health care claims data (Ministry of Health, Labour and Welfare 2014b) has resulted in the computerization of nearly all data used in insurance reimbursements (Cabinet Office, Government of Japan 2013).

Shibahara et al. (Shibahara et al. 2014) estimated the ICER of abiraterone for CRPC patients in Japan using models constructed with data from the existing literature and assumptions of general procedures. In this study, we modified these models using real-world data.

For this research, we obtained anonymized claims data of prostate cancer patients. A preliminary analysis of the database showed large variations in health care costs for CRPC patients. In particular, we found various types of chemotherapy administered to these patients. This brings the practical applicability of the previous CEAs into question, as these additional therapies were not taken into account in those analyses.

The primary objective of this study was to use real-world data to elucidate the types of treatments administered to CRPC patients in Japan after docetaxel therapy. The secondary objective was to estimate the health care costs for CRPC patients after docetaxel therapy.

## Methods

### Data source

We obtained anonymized computerized health care claims data of prostate cancer patients from Japan Medical Data Center Co. Ltd., which compiles and provides claims data from health insurance associations operated by Japanese companies. This database consists of data from more than 2.5 million insured persons aged below 75 years from over 50 companies between January 2005 and June 2013.

### Data analysis

Patients with prostate cancer were identified using recorded diagnoses. After we identified prostate cancer patients, we further narrowed the target sample by selecting patients who had been administered docetaxel; these patients were assumed to be CRPC patients who may also receive post-docetaxel treatments.

We investigated if there had been any post-docetaxel chemotherapy drugs administered to the target patients. Patients who were diagnosed with cancers other than prostate cancer were excluded from analysis as we were unable to clearly distinguish the target disease for each administered chemotherapy drug.

Health care costs were calculated per patient per month. Factors that may possibly influence costs, such as inpatient care and death, were also collected from the data. Our calculations incorporated all health care costs, including costs that were directly and not directly associated with prostate cancer. Finally, a regression model was constructed to estimate the mean health care costs of CRPC patients and to investigate the influence of each factor on these costs. The dependent variable of the regression model was health care costs per patient per month, and the independent variables included patient age, hospitalizations (inpatient care), chemotherapies, and death. Costs were calculated in Japanese yen and converted into US dollars using the purchasing power parity rate of 2013 (US$1 = ¥102.048), as provided by the International Monetary Fund (http://www.imf.org/). Statistical analyses were conducted using R software version 3.1.2, and statistical significance was set at P < 0.05. The goodness-of-fit of the model was assessed using the R^2^ value.

### Ethical considerations

This study was approved by the Ritsumeikan University Ethics Review Committee for Research Involving Human Participants (BKC-Human-2014-014).

## Results

### Prostate cancer patients

We identified 2138 prostate cancer patients from the database. From among these patients, 36 had been administered docetaxel during the study period. We excluded 15 persons who had been diagnosed with other cancers or had used docetaxel in June 2013, which was the final month of the study period. The first exclusion criterion was to focus the study on prostate cancer patients, while the second exclusion criterion enabled the analysis of post-docetaxel treatments. As a result, the final sample for analysis comprised 18 CRPC patients.

The average age of the patients in the study sample was 61.7 years (range 54–70 years). Of all the patients, 66.7 % (12 of 18 cases) were tracked throughout the entire study period, and the death of 55.6 % (10 of 18 cases) was recorded in the data.

### Post-docetaxel chemotherapy for CRPC patients

Our results showed that 66.7 % (12 of 18 cases) of CRPC patients were administered other forms of chemotherapy after docetaxel treatment (Table 1). Among these, 2 patients were administered more than one chemotherapy drug after docetaxel. The results also showed that 27.8 % (5 of 18 cases) of the patients died in the same month in which they received their last dose of docetaxel treatment.

**Table 1:** Chemotherapies administered to CRPC patients after docetaxel treatment

Drug	Number of patients (includes double counting)	%
Estramustine phosphate sodium hydrate	6	33.3
Tegafur-uracil	3	16.7
Etoposide	1	5.6
Cyclophosphamide	1	5.6
Total	8	44.4

### Health care costs

Of the 18 CRPC patients, 5 patients died in the same month in which they received their last administration of docetaxel; we investigated the health care costs of the remaining 13 survivors. The mean health care costs derived from the 67 patient-months of data from the 13 surviving CRPC patients after their last docetaxel administration was ¥223,686 (US$2183). We categorized the patients into 3 categories based on their age at the end of docetaxel use [≤59 years [5 cases], 60–64 years (4 cases), and ≥65 years (4 cases)], and included these categories in the regression model. Eight patients were found to have experienced 1 or more hospitalizations, and 6 patients were administered other chemotherapies during the study period.

The regression model to explain the health care costs used the following formula:

$$\begin{aligned} &{\text{result }} {\leftarrow}\, {\text{lm}} ( {{\text{formula }} = {\text{ costs }}\sim {\text{ inpatient }} + {\text{ chemo }} + {\text{ death }} + {\text{ age}} . {\text{c3}},{\text{ data }} = {\text{ dataset}}} )\\ & {\text{summary }}\left( {\text{result}} \right) \end{aligned}$$

where costs: health care costs; inpatient: inpatient care; chemo: other chemotherapies; death: patient death during the month of study; and age.c3: age categories.

The results of the regression model analyzing health care costs are shown in Table 2. Inpatient care and the administration of other chemotherapy drugs were significantly associated with health care costs, but age was not significantly associated with these costs. The intercept of the model showed that the mean health care costs for CRPC patients after docetaxel treatment was ¥97,506 (US$952). Hospitalization (inpatient care) increased costs by ¥333,350 (US$3790) per month, and other chemotherapy treatments increased costs by ¥126,647 (US$1236) per month.

**Table 2:** Relationship between possible factors and health care costs for CRPC patients after docetaxel treatment

Possible factors related to costs	Estimate	US$	SD	US)	P value
Intercept	¥97,506	$952	¥47,084	$460	0.04
Inpatient care	¥333,350	$3255	¥50,238	$490	<0.001
Other chemotherapy treatments	¥126,647	$1236	¥60,431	$590	0.04
Death	−¥55,100	−$538	¥79,781	$779	0.49
Age categories	¥10,439	$102	¥22,874	$223	0.65

The intercept of the model was assumed to be the mean health care cost; adjusted R^2^: 0.502

## Discussion

Using real-world data, we analyzed the actual treatments and costs for CRPC patients after docetaxel treatment. Our findings showed that 66.7 % of the study sample had been administered other chemotherapies after docetaxel. Although costs varied widely among the patients, our model estimated the mean health care costs to be ¥97,506 ($952) per patient per month. Our estimate was similar to that of a previous study (Shibahara et al. 2014), which estimated costs to range from ¥53,090 (US$518) to ¥91,060 (US$889). In addition, our analysis showed that these costs were not strongly associated with death, but were significantly affected by variations in hospitalization and chemotherapies.

It should be noted that over half of the CRPC patients in our sample had been administered chemotherapies other than those recommended in Japanese guidelines. This indicates that if applying the results of CEAs to clinical practice and policymaking, the CEAs should take into account the actual therapies provided to patients. The selection of comparison groups that used placebos in previous CEAs may therefore result in fewer practical downstream applications for those cost-effectiveness estimates. However, the authors recognize that it was reasonable for previous studies to utilize a placebo comparison group because the original clinical trial had used a similar approach, and the guidelines had not recommended other aggressive treatments. Also, the use of placebo groups may have been unavoidable due to the lack of available data. Nevertheless, the increased opportunities to investigate real-world data using current databases means that we are now able to take into account actual medical practices and identify important variables to include in evaluations.

Our finding that CRPC patients had received several types of chemotherapy may have further implications for CEAs. The use of additional chemotherapies was shown to be significantly associated with higher health care costs. Furthermore, the use of additional chemotherapies may cause complications, which can increase health care costs and reduce patient quality of life. CEAs that take the provision of additional chemotherapies into consideration may therefore lead to more favorable results for the target drugs.

There are several limitations to this study. First, the data were obtained from corporate health care insurers in Japan. Thus, this study focused on persons aged below 75 years, as persons aged 75 years or more are covered by different health care insurers. Despite the fact that a universal health care insurance system is used throughout Japan regardless of insurer, our findings may not be indicative of the entire Japanese population. Other studies are needed to include enrollees from other types of insurance systems. The upcoming Japanese national database, which includes nationwide health care claims data, has promising applications for future analyses (http://www.mhlw.go.jp/seisakunitsuite/bunya/kenkou_iryou/iryouhoken/reseputo/). Regardless, our study shows that it is highly possible that CRPC patients receive various kinds of therapies, including chemotherapies. Therefore, the actual therapies administered to patients should be taken into account when designing evaluation models. Another limitation of the study is the relatively small number of factors included in our cost analysis model. This was due in part to the small number of subjects. Although a large study sample would have been favorable, we believe that our analysis provides an acceptable estimation of costs in CRPC patients using real-world data, which has not been previously conducted. In addition, our model had a fairly high degree of goodness-of-fit. It should also be noted that the health care costs estimated here may include costs other than for the treatment of prostate cancer. Despite excluding patients who had other cancers, we did not exclude patients with other non-cancer diseases. Our estimates may therefore be higher than the actual costs for treating only prostate cancer, and it would be difficult to clearly distinguish between costs for the target disease and costs to treat complications and comorbidities using current databases.

## Conclusions

In this study, we elucidated the varieties of chemotherapies for CRPC patients that were used in actual clinical practice in Japan. We also estimated the health care costs for these treatments, and investigated the factors that may influence these costs. This study contributes to future CEAs by not only providing an estimate of health care costs for these patients, but also by demonstrating that the actual therapies provided to comparison groups should be considered when conducting CEAs for informing the policymaking process.

## References

[CR1] Cabinet Office, Government of Japan (2013) Reseputo denshika no torikumi no keii (Background of electronization of medical claim data). Homepage of Cabinet Office, Government of Japan. http://www8.cao.go.jp/kisei-kaikaku/kaigi/meeting/2013/wg/kenko/130731/item1_2.pdf. Accessed 15 Sept 2015

[CR2] de Bono JS, Logothetis CJ, Molina A, Fizazi K, North S, Chu L, Chi KN, Jones RJ, Goodman OB, Saad F, Staffurth JN, Mainwaring P, Harland S, Flaig TW, Hutson TE, Cheng T, Patterson H, Hainsworth JD, Ryan CJ, Sternberg CN, Ellard SL, Flechon A, Saleh M, Scholz M, Efstathiou E, Zivi A, Bianchini D, Loriot Y, Chieffo N, Kheoh T, Haqq CM, Scher HI, COU-AA-301 Investigators (2011). Abiraterone and increased survival in metastatic prostate cancer. New Engl J Med.

[CR3] Dyer M, Rinaldi F, George E, Adler AI (2012). NICE guidance on abiraterone for castration-resistant metastatic prostate cancer previously treated with a docetaxel-containing regimen. Lancet Oncol.

[CR4] Ministry of Health, Labour and Welfare (2014a) Hiyotaikouka hyouka senmonbukai no kongo no susumekata ni tsuite (an) (Future plan of special committe on cost-effectiveness) (in Japanese). Homepage of Ministry of Health, Labour and Welfare. http://www.mhlw.go.jp/file/05-Shingikai-12404000-Hokenkyoku-Iryouka/0000044423.pdf. Accessed 15 Sept 2015

[CR5] Ministry of Health, Labour and Welfare (2014b) Ryouyou no kyufu oyobi kouhi futaniryo ni kansuru hiyo no seikyu ni kansuru shorei (Ministerial ordinance concerning claims of expense on the provision of medical treatment and public expenditure) (in Japanese). Homepage of e-Gov. http://law.e-gov.go.jp/htmldata/S51/S51F03601000036.html. Accessed 14 Oct 2015

[CR6] Mohler JL, Kantoff PW, Armstrong AJ, Bahnson RR, Cohen M, D’Amico AV, Eastham JA, Enke CA, Farrington TA, Higano CS, Horwitz EM, Kane CJ, Kawachi MH, Kuettel M, Kuzel TM, Lee RJ, Malcolm AW, Miller D, Plimack ER, Pow-Sang JM, Raben D, Richey S, Roach M, Rohren E, Rosenfeld S, Schaeffer E, Small EJ, Sonpavde G, Srinivas S, Stein C, Strope SA, Tward J, Shead DA, Ho M (2014). Prostate cancer, version 2.2014. J Natl Compr Cancer Netw JNCCN.

[CR7] Neumann PJ, Cohen JT, Weinstein MC (2014). Updating cost-effectiveness—the curious resilience of the $50,000-per-QALY threshold. New Engl J Med.

[CR8] Shibahara H, Shiroiwa T, Tange C, Nakamura K, Ozono S, Shimozuma K (2014). Reanalysis of cost-effectiveness of abiraterone acetate as second line treatment for metastatic castration-resistant prostate cancer in japan using a japanese claim data set. Value Health.

[CR9] The Japanese Urological Association (2012) Clinical practice guidelines for prostate cancer (in Japanese). Kanehara-shuppan Press, Tokyo

[CR10] Wilson L, Tang J, Zhong L, Balani G, Gipson G, Xiang P, Yu D, Srinivas S (2014). New therapeutic options in metastatic castration-resistant prostate cancer: can cost-effectiveness analysis help in treatment decisions?. J Oncol Pharm Pract Off Pub Int Soc Oncol Pharm Pract.

[CR11] Zhong L, Pon V, Srinivas S, Nguyen N, Frear M, Kwon S, Gong C, Malmstrom R, Wilson L (2013). Therapeutic options in docetaxel-refractory metastatic castration-resistant prostate cancer: a cost-effectiveness analysis. PLoS One.

